# Supplementary material for: Are carbon emissions trading and green financial instruments synergistic? -Comprehensive quantitative research based on content analysis
Source: PLoS One. 2024 Mar 7;19(3):e0298601. doi: 10.1371/journal.pone.0298601 (PMC10919601; doi:10.1371/journal.pone.0298601)
Supplement: S1 Data — (ZIP) [file pone.0298601.s001.zip › Data/地级市Z_F工作报告.zip!/╔╜╢1⁄2/║╩╘≤╩╨/└┤╫╘╣1⁄2╓┌║┼í╛┬φ┐╦╩2╛▌═°í┐_2019.docx]

各位代表：

　　现在，我代表市人民政府向大会报告工作，请予审议。请市政协委员和其他列席会议的同志提出意见。

　　一、2018年工作回顾

　　过去的一年，面对复杂多变的外部环境，我们坚持以习近平新时代中国特色社会主义思想为指导，在市委的坚强领导下，认真贯彻党的十九大精神和习近平总书记视察山东、视察菏泽重要讲话、重要指示批示精神，深入贯彻落实中央和省委、省政府的决策部署，抢抓“突破菏泽、鲁西崛起”战略机遇，全力以赴稳增长、促改革、调结构、惠民生、防风险，经济社会发展稳中有进、持续向好。预计实现地区生产总值突破3000亿元，增长8%，增幅居全省前列；三次产业结构为9.7:51.0:39.3，服务业占比提高1个百分点；地方一般公共预算收入206亿元，增长10.4%；规模以上固定资产投资、规模以上工业增加值均增长8%；实现社会消费品零售总额1810亿元，增长10%；城乡居民人均可支配收入达到26190元和12834元，分别增长8.6%、9.2%，增幅均居全省前列。市十九届人大二次会议确定的年度目标任务圆满完成。

　　一是项目建设和“双招双引”成效明显。精心策划储备项目，44个项目列入省重点和新旧动能转换第一批优选项目，个数居济青烟三个核心区外第1位。全力推动项目落地建设，8个省重点项目完成投资50.4亿元、占年度计划的100.5%，220个市重点项目完成投资912.8亿元、占年度计划的104.3%，巨野天运新能源、东明中信国安新材料等72个项目竣工投产。坚持把招商引资作为经济工作的生命线，积极参加首届儒商大会、香港山东周，组织开展首届牡丹之都发展论坛等7次集中招商活动，全年共签约合同项目424个、总投资2146.6亿元。精进电动、复星医药、方特文化科技创意产业园等340个项目已落地实施。入选国家级示范PPP项目19个，数量居全国地级市首位，是全省唯一一个受国务院表彰的地级市。出台“人才新政30条”，柔性引进10位院士、14位海外高层次人才，海内外招才引智工作站增加到11个。分两批集中引进“双一流”高校高层次人才362名。

　　二是新旧动能转换提速增效。聚焦“七大主导产业”，全面实施新旧动能转换“十大工程”，新动能加速孕育成长。工业经济提质增效。落实工业强市战略若干政策意见，制定支持民营经济高质量发展11条，新增市场主体10.6万户，纳税过千万元企业达到112家、过亿元19家，分别增加29家和3家。生物医药产业主营业务收入持续位居全省第1位。东明石化集团主营业务收入突破千亿元大关，利税达到164.9亿元。新增省级“专精特新”中小企业14家、“隐形冠军”企业8家、“瞪羚”企业3家。阿里巴巴菏泽产业带入驻企业2506家，综合排名列全省第1位、全国第22位。东明石化产业园、定陶润鑫化工产业园等7个化工园区获省批准认定。淘汰关闭落后产能企业420家，通过“腾笼换鸟”新上了巨野世运专用汽车、成武医疗器械产业园等一批优质项目。与社会资本联合设立30亿元的新旧动能转换引导基金，助推工业转型升级。科技创新步伐加快。新增省级工程技术研究中心、工程实验室、企业技术中心21家、省级院士工作站10家，新增数量历年最多。省级以上科技创新平台达到183家。新认定高新技术企业48家、达到121家，高新技术产业产值占规模以上工业的比重达到32%、同比提高1个百分点。步长制药获得2018年度国家科学技术进步奖二等奖。新申报中国驰名商标3件，新申请马德里国际商标88件，新注册中国地理标志商标8件。开放型经济实现突破。对外投资8932.7万美元、增长432%，增幅居全省第3位。完成外贸进出口512亿元、增长26.5%，增幅居全省第2位。实际到账外资1.3亿美元、增长226%，增幅居全省第1位，其中市开发区金丰现代农业项目到位外资8000万美元。菏泽木草柳制品、鄄城人发制品、曹县家具三个基地成为“国家外贸转型升级基地”。曹县、单县被列入全省第三批经济开发区体制机制创新试点。“四新”经济加快发展。规模以上新能源新材料企业超过100家。拥有了石墨烯有机太阳能电池、高铁减震器、高端软体屏蔽材料等一批技术含量高、代表产业发展方向的新产品。山东质量链菏泽工作站挂牌成立，产品上链企业数量居全省第2位。淘宝村、淘宝镇分别发展到267个、35个，数量稳居全国地级市第1位，实现电商交易额3090亿元、增长50.7%，连续四年增幅超过50%。首届腾讯“为村”大会在我市举办。服务业提档升级。成功举办了第15届中国林产品交易会、第27届菏泽牡丹文化旅游节、第13届中国（菏泽）农资交易会暨农副产品博览会。新增省级服务业特色小镇3个、服务业产业集群1个，现代服务业增加值占服务业的比重达到52.5%。

　　三是农业农村现代化步伐加快。把乡村振兴作为 “三农”工作的总抓手，要素配置优先满足、财政投入优先保障、公共服务优先安排，整合涉农资金68亿元用于乡村振兴和脱贫攻坚。现代农业发展水平显著提升。持续深化农村集体产权制度改革，在全省率先完成清产核资任务。新增土地流转面积108.4万亩、流转率达到33.1%，土地托管面积达到425万亩，新增家庭农场省级示范场11家。加快发展农业“新六产”，20个现代农业产业综合园区完成投资153.4亿元，规模以上农副产品加工企业达到1833家，农产品加工转化率提高2个百分点。大力发展特色农业、品牌农业，新增国家级“一村一品”示范村镇3个、达到14个，新增省知名农产品品牌8个、“三品一标”产品105个。狠抓农产品质量安全，新增省级以上农产品质量安全县6个。“菏泽牡丹”被评为山东省区域公共品牌，巨野大型牡丹画《花开盛世》《锦绣春光》“绽放”上合组织青岛峰会和首届中国国际进口博览会，扩大了菏泽牡丹的世界影响力。鄄城入选全省乡村振兴“十百千工程”示范县。农村环境明显改善。创新实施农村新型社区和新型小城镇建设融合发展，启动试点乡镇16个，创建国家级特色小镇5个、省级12个。大力实施农村“七改”工程，改造农村危房4507户，改厕35万户，新修改造农村公路5213公里。积极推进农村全域保洁市场化，村容村貌焕然一新。深入推进国家森林城市创建，新增造林面积38万亩，造林质量、数量创历史新高。乡村文明行动深入推进。大力弘扬优秀乡村文化，建设乡村记忆博物馆35个，建成乡镇综合文化站154个。积极创新乡村治理模式，扎实推进殡葬综合改革，移风易俗持续深化。

　　四是城市面貌显著改观。坚持以大投入推进大建设，城市公共服务设施、道路交通环境、生态宜居水平实现了量的大扩容和质的大提升。重大交通工程加快实施。菏泽牡丹机场具备全面开工条件，鲁南高铁曲阜至菏泽段启动建设，雄商高铁菏泽段可研报告正在编审、有望今年上半年开工，菏徐城际铁路菏泽段被纳入省综合交通网发展规划，全市人民期盼多年的“航空梦、高铁梦”越来越近。德上高速、枣菏高速菏泽段建设加快推进，日兰高速巨野至菏泽段改扩建工程启动，七条国省道改扩建工程集中实施，新万福河复航工程快速推进，立体化大交通网络正在形成。公共服务设施更加完善。市民文化中心主体基本完成，市体育公园、市档案馆、市展览中心、环堤公园等一批城建重点工程启动建设。建成综合管廊10公里、海绵城市18.4平方公里，铺设燃气管道62公里、供热管网73公里，城市公共服务功能不断增强。城市承载能力显著提升。完成了“四区”区划调整，出台了支持“四区”加快发展的意见，城市发展更加协调。大力推进棚户区改造，完成12.7万套棚改任务。着力拉开城市发展框架，打通断头路33条，新修市区道路41条、84.6公里，基本完成中华路、黄河路、牡丹路等12条骨干道路升级改造，渠化路口43个，市区路网日益通畅。县城建设各具特色，郓城规划建设呈现大气魄，成武水城结合文章做得好，巨野公共服务设施配套完善，单县城市管理市场化水平高，等等。单县、鄄城被列为省级新型城镇化试点，鄄城入选全省农村宅基地“三权分置”改革试点。城市精细化管理水平明显提升。以国家文明城市创建为抓手，大力推行网格化管理，深入开展市区超标电动车、违法建设、占道经营、户外广告等城市管理“十项整治”行动，城市更加整洁有序，宜居水平明显提升。按照“10个有利于”的要求，经过几年的不懈努力，现在的菏泽城市一片片破旧不堪的棚户区快速消失，一个个配套完善的新社区如雨后春笋般不断涌现，城市的环境更优了、形象更靓了、活力更强了，一座现代化的区域性中心城市呼之欲出！

　　五是可持续发展基础更加稳固。聚焦“三大攻坚战”，全力补短板、防风险、促稳定。脱贫攻坚取得历史性突破。持续深化“三个一”工程，投入专项扶贫资金19.4亿元，实施产业扶贫项目1050个。在全省率先实施“大病医疗商业补充保险+小额人身意外伤害保险”，发放保险金3.4亿元，有效破解了因病致贫返贫的难题。聚焦聚力黄河滩区脱贫迁建，28个村台、6个外迁社区全部开工，其中18个村台完成吹沙淤填，5个外迁社区主体完工，近千名群众春节前将喜迁新居。全省电商兴农扶贫大会、全省乡村振兴暨脱贫攻坚西片区现场会、全省电力扶贫开发工作推进会在我市召开。经过全市上下戮力攻坚，脱贫任务基本完成，共有170.15万名群众甩掉了贫困的帽子，加快了致富奔康的步伐。风险防范化解有力有效。以防范金融风险为重点，全力开展“破圈断链”行动，帮助一批企业渡过了难关。严厉打击非法集资、恶意逃废债行为，金融风险总体可控。扩充企业融资增信平台资本金，市金和融资担保公司财政注资规模达到20亿元，居全省各市首位。菏泽牡丹信用保险股份公司已报银保监会审批。认真贯彻房地产调控政策，严格落实“房住不炒”原则，加强房地产市场监管和隐患排查，及时应对市场变化，促进了全市房地产市场平稳发展。生态环境质量持续改善。坚持“绿水青山就是金山银山”的发展理念，全面推进“四减四增”行动，全力打好“九场标志性战役”，扎实做好中央和省环保督察反馈问题整改，集中解决了一批环境突出问题，中央环保督察“回头看”交办我市信访件数量全省最少。探索实施“路长制”，全面推行国省道市场化保洁，路域环境明显改善。持续推进煤炭清洁化利用，完成“双替代”7.6万户，被列为第二批全国冬季清洁取暖试点城市。全年PM2.5平均浓度改善率全省第1位，空气优良天数达到191天、同比增加24天，增加天数列全省第1位。新建、扩建污水处理厂17座，新铺设污水管网471.6公里，三条主要河流出境断面水质全面达标，入选2018年全国黑臭水体治理示范城市，曹县黄河故道湿地公园被命名为“国家湿地公园”。社会形势持续稳定。持续推进安全生产风险分级管控和隐患排查治理双重预防体系建设，扎实开展“大快严”集中行动，全市生产安全事故起数、死亡人数实现双下降。民兵调整改革任务圆满完成，国防动员能力进一步增强。创新社会治安综合治理，深入开展“扫黑除恶”专项斗争，大力铲除人民群众深恶痛绝的黑恶势力、“保护伞”，有力维护了全市社会安全稳定。

　　六是民生保障更加有力。牢固树立以人民为中心的发展思想，主动回应群众关切，积极兴办民生实事。全市民生支出441亿元、增长9.4%，占一般公共预算支出的80%。教育短板加速补齐。投入资金40.6亿元，完成“全面改薄”项目338个，新建改扩建城区中小学141所、幼儿园279所，新增中小学学位9.4万个、幼儿园学位3.6万个，开工建设乡村教师周转宿舍项目53个，新招聘教师4506人。基础教育质量稳步提升，义务教育由基本均衡迈向优质均衡的新阶段。市职教园区启动建设，职业教育迈出新步伐。社保体系不断完善。全面落实就业创业扶持政策，新增城镇就业7.8万人，城镇登记失业率控制在3.05%。基本养老保险参保人数达到578.2万人，实现了应保尽保。城市低保月保障标准和农村低保年保障标准分别提高到440元和3800元。新建、扩建养老机构18处，新增养老床位4200张，社区居家养老服务经验在全省推广，入选全国居家养老和社区养老服务改革试点城市。健康菏泽加快推进。省级食品安全先进市县实现全域创建，人民群众饮食用药安全保障更加有力。全市医养结合机构发展到52家，196家医疗卫生机构参与组建医联体，域外转诊率逐步下降。万名残疾儿童康复工程深入实施。文体事业蓬勃发展。省级文明城市创建成功，全国文明城市创建扎实推进，市民文明意识和城市文明程度明显提升。积极发展文化产业，菏泽广电传媒集团、菏泽文旅集团挂牌成立。村级基层综合性文化服务中心实现了全覆盖，建成农家书屋4200余家。定陶区两夹弦非遗保护传承中心被评为第七届全国服务农民、服务基层文化建设先进集体，牡丹区、巨野、郓城被评为第三届山东省文化强省建设先进县。全市体育活动蓬勃开展，“村级全民健身全民健康十个一工程”经验全省推广。同时，双拥优抚、人民防空等工作得到新提升，民族宗教、外事侨务、统计审计、气象地震、档案史志及妇女儿童、关心下一代、老龄、红十字等各项工作取得新成效。去年确定的十个方面的民生实事基本完成。免费孕前健康检查实现了目标人群全覆盖，优生优育水平明显提高；新建市区 “口袋公园”100处，方便了市民生活；新建改造市区污水管网138公里，有效提升了污水收集处理能力；新建城市社区老年人日间照料中心25处、农村幸福院293处；完成农村公路“户户通”工程1500公里；开通了市区至各县的城际公交，大大方便了群众出行，等等。

　　七是政府履职服务能力进一步提升。全力促改革、转作风、建机制，行政效能明显提升。思想解放持续深入。深入开展“大学习、大调研、大改进”，组织到省内和江浙等先进地区考察学习，以思想观念转变引领创新发展、激发内在动力。法治意识更加牢固。严格执行重大事项决策程序，积极推行行政执法“双随机一公开”制度。依法接受人大工作监督，自觉接受政协民主监督，主动接受社会监督和舆论监督。办理省市人大代表建议132件、政协提案411件，满意率均达100%。工作作风务实高效。将260项重点工作分解落实到牵头领导、责任单位和责任人，完成情况定期向社会公开。面对棚改拆迁、市区城市管理“十项整治”、治污治霾、防范化解金融风险、违法用地整治“百日攻坚”、“三城同创”等艰难繁重的工作任务，各级各部门知难而进、迎难而上，全面完成了各项任务目标，树立了务实担当、干事创业的良好形象。政务服务明显优化。全面启动政府机构改革，市政府部门完成组建并按新体制运行。围绕深化“放管服”改革，着力推进“一次办好”，积极开展优化营商环境11个专项行动。创新实行市、县领导到政务服务中心值班制度，现场解决群众反映问题，得到了省委主要领导的批示肯定。新组建市、县两级行政审批服务局，实行“一枚印章管审批”，市级239项行政许可事项及91项关联事项已全部划转到位。全面推行贴心帮办制、错时延时制、“容缺审批”制等，最大限度方便企业和群众办事，群众满意度大幅提升。廉政建设持续深化。严格落实党风廉政建设“一岗双责”，持之以恒正风肃纪，把全面从严治党贯穿到了政府工作各方面。严格遵守中央八项规定精神，驰而不息纠正“四风”，严防反弹回潮。深化领导干部经济责任审计，全面开展“落实党风廉政建设主体责任”廉政谈话，深入推进反腐倡廉工作，打造了风清气正的政务生态环境。

　　过去的一年是全市上下奋力拼搏、攻坚克难的一年，也是在“决胜全面小康、实现后来居上”征程中，取得重大成效的一年。成绩的取得，是习近平新时代中国特色社会主义思想科学指引的结果，是省委、省政府和市委正确领导的结果，是市人大、市政协监督支持的结果，是全市广大干部群众苦干实干、奋力拼搏的结果。在此，我代表市人民政府，向全市人民，向各级人大代表、政协委员，向各民主党派、工商联、人民团体和社会各界人士，向离退休老领导、老同志，向驻菏部队、武警官兵、公安干警、消防救援队伍，向所有关心支持菏泽建设和发展的同志们、朋友们，表示衷心的感谢并致以崇高的敬意!

　　同时，我们也清醒地认识到，全市经济社会发展中还存在不少困难，政府工作也存在许多不足。经济总量较小，产业结构不合理、科技创新能力弱等问题依然突出；“四新”经济总量不够大，加快新旧动能转换、实现高质量发展的任务艰巨繁重；县域经济发展不平衡、同质化等问题仍然存在；金融风险隐患依然存在，巩固脱贫成果、生态环境建设、安全生产等面临较大压力；基础设施、公共服务等民生领域欠账较多，教育、医疗、社会保障等方面还存在很多短板；职能转变还不到位，有些政府部门和工作人员还存在不担当、不作为、形式主义、官僚主义等作风问题，政务服务质量和效率与企业和群众的期盼还有很大差距。对此，我们一定将以对人民高度负责的担当精神，采取更加有力有效的措施，认真加以解决。

　　二、2019年经济社会发展的总体要求和任务目标

　　今年是新中国成立70周年，是全面建成小康社会的关键之年，做好今年的政府工作意义重大。

　　分析当前面临形势，国内外经济环境严峻复杂，下行压力没有根本缓解，市域经济仍处于结构的深度调整期、瓶颈的突破期和动能转换的胶着期。同时也深刻认识到，深入推进高质量发展、实现后来居上，我们面临的机遇前所未有。从宏观环境看，在“六稳”的主基调指引下，国家加大重大基础设施建设推进力度，通过定向降准等措施推动更多资金向实体经济集中，持续深化减税降费、“放管服”改革等支持创业创新，这些与我市密集实施的重大基础设施建设、加快新旧动能转换、解决企业融资难题等重点工作高度契合。从我市基础看，近年来全市上下牢牢把握高质量发展要求，在重点项目建设、“双招双引”、乡村振兴等方面集中发力，形成了多点突破的良好局面。从政策支撑看，我市相继纳入中原经济区、山东西部经济隆起带和淮河生态经济带发展规划。特别是去年9月，省委、省政府出台《关于突破菏泽、鲁西崛起的若干意见》，在产业培育、基础设施建设、转移支付、创新发展等方面给予全方位支持，政策力度之大、覆盖面之广、含金量之高前所未有，为我市推进高质量发展提供了难得的历史机遇。

　　各位代表，机遇稍纵即逝，时不我待，我们将牢牢抓住、用心研究，充分利用、借势发力，全力以赴补短板、塑优势、提质效、增潜能，全力打造鲁西崛起的高地、全省新旧动能转换的示范区、鲁苏豫皖四省交界的区域性中心城市，切实把机遇转化为推动高质量发展的现实生产力，努力把省委、省政府的关心厚爱转化为突破菏泽、加快发展的强劲动力，早日把习近平总书记提出的“后来居上”的殷切期望变成生动美好的现实！

　　今年政府工作的总体要求是：坚持以习近平新时代中国特色社会主义思想为指导，认真学习贯彻党的十九大和十九届二中、三中全会精神，深入贯彻落实习近平总书记视察山东、视察菏泽重要讲话、重要指示批示精神，全面贯彻中央和省委、市委决策部署，抢抓“突破菏泽、鲁西崛起”战略机遇，围绕“后来居上”奋斗目标，牢固树立新发展理念，坚持以提高发展质量和效益为中心，以深化供给侧结构性改革为主线，以新旧动能转换“十大工程”为抓手，聚焦“七大主导产业”、新型城镇化、乡村振兴、民生保障等重点，继续打好“三大攻坚战”，做好“六稳”文章，深化市场化改革，扩大高水平开放，促进经济社会持续健康发展。

　　今年全市经济社会发展主要预期目标为：地区生产总值增长7.5%左右；地方一般公共预算收入增长8%左右；固定资产投资增长8%左右；社会消费品零售总额增长9%左右；城乡居民人均可支配收入分别增长8%、8.5%左右；常住人口城镇化率提高1个百分点左右，城镇登记失业率控制在3.5%以内。全面完成省下达的节能减排降碳约束性指标和环境质量改善目标。

　　三、2019年政府工作重点任务

　　今年经济社会发展任务十分繁重，必须科学谋划、突出重点、统筹兼顾，全面抓好各项工作落实。

　　（一）全力推动新旧动能转换重大工程，着力推进经济高质量发展。坚持把新旧动能转换“十大工程”作为经济工作主抓手，努力以“四新”促“四化”，不断增创高质量发展新优势。一是突出抓好重点项目建设。继续坚持重点项目集中开工、领导干部包保、月调度、半年现场观摩等推进机制，建立管理服务平台，对重点项目进展情况实时监控、跟踪服务。优选150个市重点项目，完成投资600亿元以上。积极搞好对接，力争大唐电力、东明石化精深加工园区、单县清控润康高端智慧服务机器人、鄄城华斯壮能源科技等重大项目落地建设。二是持续加大“双招双引”。把“双招双引”作为全局性、根本性、关键性工作，以招大引外、招新引高为主攻方向，突出抓好专业招商、社会化招商、产业链招商和园区招商，盯紧国际国花博览园、伏羲智库、城际空间站等一批重大项目，由市级领导包保，建立联席会议制度，抓好推进落实，确保新签约落地过亿元项目220个以上，其中过10亿元项目15个以上。深化人才“第一资源”意识，全面落实“人才新政30条”，用好招才引智工作站，深化与高等院校和科研院所的战略合作，建好非物质文化遗产研究院、菏泽·来投吧、山东高端化工研究院等人才创新平台，力争引进高层次人才300名。加强智库建设，聚集社会智力资源，推动创新发展。坚持政策引导，用心用情服务，全年新增返乡创业就业6.5万人。三是做大做强主导产业。落实“七大主导产业”发展规划，着力打造一批千亿级产业集群。鼓励县区立足优势、错位发展，大力培育各具特色的主导产业。牡丹区重点发展商贸物流和电子商务产业，市开发区重点发展新能源和新装备产业，市高新区重点发展生物医药和新材料产业，东明、郓城、巨野重点发展高端化工产业，定陶区、鄄城重点发展生物医药产业，曹县、单县、成武重点发展现代农副产品加工、电子商务、机电设备制造等产业。推进重点产业建链拓链壮链，加快技术创新、管理创新、商业模式创新，培育一批行业领军企业，推动主导产业扩规模、上水平、增效益。实施品牌带动战略，着力推进标准化改革试点重点项目，培育中国驰名商标2件、中国地理标志商标6件，申请马德里国际商标30件，进一步提升产业发展影响力和竞争力。围绕产城融合，完善产业园区发展规划。大力推进园区体制改革，以去行政化为方向，探索“管委会＋公司”模式，实行灵活的用人制度和薪酬体系，让开发区“回归本源”。进一步推动市开发区、市高新区加快建设国家级园区。四是加快推进科技创新。强化科技创新政策引导，加大多元化科技投入，年内全社会研发投入占GDP的比重达到1.5%。搭建科技创新载体，加快推进省科学院菏泽分院、省生物工程技术创新中心建设，积极筹建菏泽产业技术研究院、中科控股技术协同创新中心、山东大学药物非临床安全性评价中心等重大创新平台，新建重点实验室、工程技术研究中心、院士工作站等国家和省级研发机构10家以上。实施高新技术企业数量、质量双提升工程，力争新认定高新技术企业40家以上，高新技术产业产值占规模以上工业的比重提高1个百分点。打造“政产学研金服用”协同创新共同体，建设山东中原技术交易市场，年内推动100家企业与高校院所开展产学研合作，转化重要科技成果100项以上。

　　（二）加快区域中心城市建设，着力提升城市载体功能。围绕打造“本地人留恋、外地人向往”的区域性中心城市，加快完善城市功能配套，不断提升城市建设管理质量和水平。一是努力提升中心城市能级。坚持规划引领，全面完成《菏泽市城市总体规划（2018-2035年）》等空间规划和各类控制性详细规划。以机场、高铁建设为依托，启动高铁新城、万福新城规划建设，实现“菏定一体化”发展，推动城市发展逐步由“赵王河时代”跨入“万福河时代”。以环堤公园建设为契机，加快推进城市“双修”试点和古城保护开发，加快实施赵王河一期公园景观提升和赵王河下游、万福河、思源河、洙水河湿地等建设，完成城区重点绿化工程，彰显花城水邑特色。以曹州牡丹园升级改造和体育公园、国花博览园、方特文化科技创意产业园等项目建设为重点，打造北部文旅高地。以完善城市功能为重点，快速推进市民文化中心、市展览中心、鲁西南大数据中心、菏泽商务中心、新闻大厦、菏泽文化广场、新博物馆、新客运站等重点项目，建成一批城市精品工程。以构建内联外通的交通网络为目标，加快建设上海路跨万福河、人民路与南外环互通立交、北外环下穿京九铁路等城市重点桥梁6座，新建提升北外环路（人民路至昆明路）、长江路（上海路至机场）、昆明路（新石铁路至国花大道）等城市骨干道路8条，进一步提升城市的通透性和承载力。启动高新区魏楼水库和城区第二水厂建设，增强市区供水能力。加快菏泽1000千伏特高压及配套工程建设，不断满足全市电力增长需求。二是加快建设交通枢纽城市。把重大交通项目作为建设区域性中心城市的重要支撑。全力推进10项在建交通重点工程，菏泽牡丹机场达到试飞条件，菏宝高速东明黄河公路大桥建成通车，德上高速巨野至单县段、枣菏高速菏泽段等具备通行条件，鲁南高铁菏泽至曲阜段、日兰高速巨野西至菏泽段改扩建工程等加快建设。启动推进鲁南高铁菏泽至兰考段、雄商高铁菏泽段、德单高速东阿至郓城段等10项交通重点工程，全面做好菏泽至徐州城际铁路、菏泽至濮阳城际铁路、东鱼河航道等10项交通重点工程前期工作，努力构建“市内大循环、外围大辐射、区域大联通”的现代化立体交通网络。三是大力推进新型城镇化。立足增强城镇的吸引力和集聚力，全力推进菏泽大城市、郓城中等城市、东明Ⅰ型小城市试点，扎实推进郓城国家级和鄄城、单县省级新型城镇化试点建设，积极做好县城和重点镇规划建设，力争实现“三个市民化”21万人。持续推进棚户区改造，完成棚户区改造3.2万套、500万平方米，不断改善居民生活环境。深入开展“全市安置房建设推进年”活动，交付使用和基本建成安置房13万套，尽早让群众回迁安居。四是切实提升城市管理水平。坚持精细化管理，全力推进全国文明城市、国家卫生城市创建。积极推进城市管理市场化改革，引入竞争机制，实行“管办分离”“管养分离”。继续深化城市管理执法体制改革，着力构建“权责明晰、服务为先、管理优化、执法规范”的城市管理体制，促进城市高效有序运行。积极推进智慧城市建设，探索运用大数据、云计算，推动城市管理向个性化、精细化、数字化、智能化方向转变。全力巩固市区城市管理“十项整治”行动成果，深入开展“城市品质提升三年行动计划”，努力让城市有内涵、有品位、有温度！

　　（三）大力实施乡村振兴战略，着力提高农业农村现代化水平。坚持农业农村优先发展，加大涉农资金整合力度，助力乡村振兴和脱贫攻坚，持续推进农业更强、农村更美、农民更富，努力探索乡村振兴齐鲁样板的菏泽路径。一是加快发展现代农业。大力发展规模农业，抓住土地流转这个关键，引导土地向农业大户、家庭农场和龙头企业集中，新增土地流转面积50万亩以上。稳定和优化粮食生产，确保粮食安全。加快发展高效农业，努力把“吨粮田”变成“万元田”。着力发展品牌农业，新增省知名农产品品牌4个以上、“三品一标”产品100个以上。积极发展农业“新六产”，加快推进20个现代农业产业园区建设。推进农村电商提档升级，培育“互联网+农业”优势品牌30个以上，淘宝村镇分别达到300个、40个，牢牢守住全国领先地位。大力推进中药材规模化种植、标准化生产，完善产业链条，助力打造“中国北方医药城”。认真落实牡丹产业发展规划，建设中国牡丹谷、举办首届世界牡丹大会和国际牡丹文化旅游节，擦亮“牡丹之都”品牌。二是提升村镇发展水平。大力推进“两新”融合发展，抓好16个试点小城镇建设，为新型城镇化打造样板。依托产业特点、文化底蕴和生态禀赋，打造一批富有活力的特色小镇。扎实推进美丽乡村建设，高标准完善功能配套，新打造省级美丽乡村100个、市级美丽乡村片区30个以上。完成土地“增减挂钩”2万亩以上。三是打造生态宜居环境。深化农村“七改”工程，新增农村清洁取暖7万户。开展饮水安全两年攻坚行动，农村自来水普及率达到95%以上。深入推进农村人居环境整治三年行动，完善农村环卫设施，大力推行垃圾分类处理。启动农村污水治理试点，探索改厕与生活污水治理一体化模式。深化路域环境综合治理，进一步完善“路长制”，实现公路保洁、绿化市场化。大力发展农村“边角经济”，充分利用房前屋后、沟渠路旁等闲散土地，开展林果种植等多种经营，促进集体增收、村庄增色。加大国家森林城市创建力度，新增造林绿化面积35万亩以上，基本完成“创森”任务。四是加强乡村社会治理。积极推进“雪亮工程”建设，深化农村网格化服务管理，扩大“一村一警务助理”覆盖面，提升“一村一法律顾问”服务质效。严格落实“四议两公开”制度，保障村级事务规范有序运行。进一步深化移风易俗，巩固提升殡葬改革成果，推行健康文明生活方式，营造良好的社会风尚。

　　（四）全面深化改革开放，着力增强发展的动力和活力。坚持以更大力度、更快节奏、更宽范围推动改革开放再出发。一是持续加大改革力度。坚持市场化改革方向，深化国资国企改革，支持市属国有投资公司健康发展。积极推进金融改革，推动农商银行业务逐步回归本源，加快推动设立菏泽牡丹信用保险股份公司、东明石化财务有限公司，启动民营银行筹建工作，加大域外金融机构引进，与省财政厅联合设立规模50亿元的“山东菏泽新动能基金”，吸引更多金融资本和社会资本投入我市。深化公共资源市场化配置改革，不断扩大政府购买服务范围。全面实施预算绩效管理，积极推行“四本预算”和政府债务收支计划一体化编制。加快落实税制改革举措，完善税收征管办法。全面完成市县乡政府机构改革任务，构建系统完备、科学规范、运行高效的行政体系。坚持以“一次办好”改革为关键抓手，扎实推进减证便民、证照分离、流程再造等，全力打造“少高优强”的营商环境。二是不断扩大对外开放。加快陆港产业新城建设，推进济铁物流园区海关监管场站、菏泽保税物流中心（B型）申报建设，多渠道搭建优质对外开放平台。进一步完善外经贸会商机制，引导企业积极参加广交会、华交会等境内外展会，多元化开拓国际市场。大力发展跨境电商，鼓励有条件的企业到主要出口市场设立海外仓，搭建以海外仓为支点的目的国配送辐射网点。认真落实国家、省促进外贸稳定增长的政策措施，加大服务贸易、高新技术和机电产品企业出口支持力度，实现对外贸易额稳步增长。充分利用香港山东周、日韩山东商务周等平台，拓宽利用外资领域，力争实际利用外资增长10%以上。三是大力支持民营经济发展。全面落实支持骨干企业、创新型企业、高成长小微企业等非公有制经济发展的奖补政策，助推各类民营企业快速成长。建立健全政银企协作机制，抓好市县担保公司运行，大力推行无还本续贷、专利质押融资、规范贷款担保等措施，合力破解民营企业融资难、融资贵问题。坚持无需不扰，采取设立“企业宁静日”、提升检查专业化水平、实行行政处罚预警制度等措施，为民营企业发展提供宽松环境。加强企业人才引进，抓好企业家培训，不断提升民营企业的后劲和活力。加强对企业家的关爱和保护，让他们在社会上有地位、政治上有荣誉、事业上有保障。

　　（五）坚定不移守住发展底线，着力巩固和谐稳定大局。牢固树立底线思维、红线意识，扎实做好防风险、补短板等各项工作。一是强化生态环境保护。坚持绿色发展理念，深入推进“四减四增”三年行动，着力打好“九场标志性战役”，重拳治理大气污染，突出攻坚PM10，确保各项大气指标持续改善。以污水收集处理、河道综合整治、黑臭水体治理为重点，加大水污染治理力度，确保三条主要出境河流断面水质稳定达标。加大农村面源污染整治，持续改善土壤环境质量。严格落实企业主体责任，加快完善污染防治长效机制，健全生态环境损害赔偿、排污许可、环保信用评价等制度。充分发挥监管网络和智慧化监管平台的作用，全面提升生态环境监管水平。增强服务意识，帮助企业制定环境治理解决方案，避免处置方式简单化、一刀切。二是织密安全生产网络。树牢安全发展理念，严格落实安全生产责任，加强应急工作，推进安全风险分级管控和隐患排查治理双重预防体系全覆盖，全面夯实安全生产基层基础。持续推进危化品综合治理和化工产业转型升级，加快企业进区入园步伐。突出道路交通、煤矿、危险化学品、建筑施工、特种设备等重点行业领域的专项治理，推进安全管理专业化、信息化、智能化，坚决防范和遏制重特大安全事故发生。加强农村安全文化教育宣传，建设一批安全教育体验基地和安全文化主题广场，全面普及安全知识，确保全市安全生产形势持续稳定。三是防范化解债务金融风险。有序处置存量政府债务，规范政府举债行为，妥善解决在建公益项目后续融资。强化金融风险监测预警，努力把风险隐患消灭在萌芽状态。深入推进“破圈断链”，严厉打击恶意逃废银行债务行为，力争银行不良贷款率降至全省平均水平，打造良好金融生态环境。四是切实加强土地保护和集约节约利用。加强永久基本农田保护，实施耕地质量保护与提升行动，积极推进基本农田储备区建设。加强土地执法监察源头防控，实施网格化管理，严厉查处各类违法违规用地行为。持续强化“亩均论英雄”的理念，加大对投资强度、亩均收益的硬约束。坚持用好增量与挖潜存量相结合，力争年内消化利用批而未供和闲置土地1万亩以上。五是全力维护社会和谐稳定。完善应急管理和预警信息发布体系，提高突发事件救援处置能力。深入开展扫黑除恶专项斗争，严厉打击黑拐骗、黄赌毒等违法犯罪活动。借鉴“枫桥经验”，推进城乡社区治理和服务创新。加强矛盾纠纷排查，改进信访工作，及时疏导和化解社会矛盾。严格落实各项政策，加强教育管理服务，维护退役军人合法权益。进一步做好民族工作，构建积极健康的宗教关系。

　　（六）大力保障改善民生，着力满足群众美好生活需要。人民对美好生活的向往就是我们的奋斗目标。持续加大民生投入，全力提升基本公共服务水平，倾情倾力做好各项民生工作。一是大力巩固脱贫攻坚成效。保持各项扶贫政策稳定，统筹各类扶贫资源，不断提升贫困人口“三保障”水平。坚持脱贫攻坚与乡村振兴融合推进，注重发展长效扶贫产业，推进扶贫车间转型升级，健全贫困群众稳定增收长效机制，推动由稳定脱贫向逐步致富转变。把黄河滩区脱贫迁建作为打赢脱贫攻坚战的重中之重，把质量安全作为百年大计，聚集各方力量，努力实现“一村一品、一台一韵”，建成美丽乡村示范点，逐步将“黄河滩”变成美丽的“花果园”。二是继续优先发展教育事业。深入实施第三期学前教育三年行动计划和2018-2020年全面消除义务教育学校大班额规划，多渠道扩充普惠性学前教育资源，基本消除“大班额”。深入推进校长职级制度改革，继续推动中小学教师“县管校聘”制度。实施高中阶段教育普及攻坚计划，推动教育向优质均衡发展。强化中高等职业教育，支持技师学院、家政职业学院全面发展，启动建设菏泽职业学院二期，市职教园区建成投用，努力打造区域职业教育发展高地。鼓励社会力量提供多样化教育服务。支持菏泽医专新校区建设，支持菏泽学院筹建菏泽大学、齐鲁工业大学菏泽校区扩大办学规模。加强师德师风建设，弘扬尊师重教社会风尚。三是全面提高社会保障水平。把稳就业摆在突出位置，落实就业优先战略和积极就业政策，重点解决好高校毕业生、农民工、退役军人、就业困难人员等群体就业。保持城镇零就业家庭“动态清零”。健全终身职业技能培训制度，年内培训5万人以上。全面实施全民参保计划，实现“社保惠民工程”全覆盖。进一步提高城乡居民最低生活保障和特困供养标准，做到应保尽保。认真落实困难家庭儿童、老年人、残疾人福利制度，健全农村留守儿童、老年人关爱服务体系。建立市残疾人服务中心，持续推进万名残疾儿童康复工程。扎实开展医养结合示范城市创建，加快推进市中医院医养结合等项目建设，创建市级示范单位20个以上。完善居家和社区养老政策法规体系，实现居家养老服务全覆盖。加强中心敬老院管理，打造农村区域性养老服务中心，全面提升社会化养老水平。四是扎实推进健康菏泽建设。做好国家卫生城市、省市卫生县城（村镇）创建工作。继续深化医药卫生体制改革，加强医疗、医保、医药“三医联动”，加快完善分级诊疗制度，促进医疗资源下沉。支持市立医院、市第三人民医院扩建新建。支持中医药创新发展，积极申报国家中医药综合改革试验区。全面落实市委《加强基层医疗卫生事业发展12条意见》，提升基层医疗卫生机构服务能力。完成省级食品安全先进市县创建。继续实施国家“学生饮用奶计划”，启动中小学生营养午餐配送工程，不断改善学生营养状况和身体素质。大力开展群众性体育活动，全面推进“村级全民健身全民健康十个一工程”，促进人民群众身体素质不断提升。五是大力推动文化旅游繁荣发展。深入挖掘厚重文化资源，加快文化强市建设。推动乡镇标准化综合文化站建设，完善村级基层综合性文化服务中心设施，积极打造乡村记忆博物馆示范点，举办好冬春文化惠民季等品牌活动，继续做好送戏下乡，进一步丰富群众文化生活。推进非物质文化遗产保护传承和历史文化遗址的保护修复，加快申报国家级曹州文化生态保护试验区，进一步提升文物保护利用水平。深入开展文化市场领域专项整治行动，切实净化社会文化环境。支持郓城水浒好汉城打造国家5A级景区，推进牡丹文创小镇、菏泽华夏部落文化旅游区等重点项目建设，推动中医药健康、休闲观光、文化体验等各类新业态旅游产业发展。继续推进旅游厕所革命新三年建设计划。办好跑游山东2019菏泽牡丹国际马拉松、菏泽（东明）黄河生态马拉松、海峡两岸第七届中华传统武术文化交流大赛等体育赛事，做好体育和旅游结合的文章。六是抓好10个方面的民生实事。（1）启动市区智慧停车系统平台建设，建成智慧停车泊位4000个。（2）新建农村公路3000公里，改造窄危桥100座。（3）新增市区新能源公交车100辆，县城到乡镇全部通公交，65%的建制村通公交。（4）新增市区公共自行车3000辆。（5）新建市区高标准公厕50座。（6）新建市区便民农贸市场6个。（7）新增城乡义务教育学校100所，新建幼儿园160所，建设高中阶段学校10所。招聘中小学教师3800名。（8）新建社区养老服务中心26处。（9）提升乡镇医院50家、村卫生室1000家。（10）新建供销社为农服务中心30个，村社共建村达到1000个。各位代表，群众的事情无小事，一枝一叶总关情。我们将时刻把群众的冷暖安危放在心上，扎实办好民生实事，努力让全市人民生活得更有尊严、更为舒心、更加幸福！

　　四、全面加强政府自身建设

　　　高质量发展需要高效能政府、高素质干部。顺应人民新期待，开启发展新征程，必须秉持务实、创新、高效、优质、担当的工作理念，坚定不移加强政府自身建设。

　　（一）强化政治建设，践行新使命。切实增强“四个意识”，坚定“四个自信”，做到“两个维护”，自觉强化政治责任，始终同以习近平同志为核心的党中央保持高度一致。坚定践行习近平新时代中国特色社会主义思想和习近平总书记视察山东、视察菏泽重要讲话及重要批示指示精神，坚持把党的领导贯彻和体现到政府工作各领域全过程，以实际行动确保党中央、国务院政令畅通，确保省委、省政府和市委各项决策部署落地生根见效。全面落实意识形态工作责任制，不断增强工作凝聚力和引领力。

　　（二）强化能力建设，提升新本领。坚持全面提升政府工作人员的专业素养、专业能力，着力增强领导干部“八个本领”，打造一支定力深、眼力准、能力强、合力好的高素质干部队伍。全面培养“挖掘金矿”的本领，瞄准国家最新产业政策，着眼全省加快新旧动能转换重大工程，抢抓“突破菏泽、鲁西崛起”重大机遇，善于从政策中发现机会、寻找资金、谋划项目，推动全市经济高质量发展。按照省委、省政府“工作落实年”的要求，不断提升狠抓落实的本领，围绕全市改革发展大局，把各项工作向深处、向细处、向实处推进。大力弘扬“勤勉敬业、担当实干、马上就办、持之以恒”的工作作风，切实增强抓落实的狠劲、巧劲和韧劲，勇于攻坚克难，以钉钉子精神做好政府各项工作。

　　（三）强化依法行政，体现新要求。积极推进法治政府建设，切实做到法无授权不可为、法定职责必须为。自觉接受人大法律监督、工作监督和政协民主监督，认真听取人大代表、政协委员，民主党派、工商联、无党派人士和各人民团体意见，主动接受社会监督和舆论监督。坚持科学、民主、依法决策，对涉及重大公共利益、群众切身利益的事项，主动听取各方面意见，真正使政府工作体现人民意愿。加强审计工作，规范公共资源交易和政府采购，强化政务公开，推进行政权力事项全部上网，确保权力在阳光下运行。

　　（四）强化使命担当，展现新作为。牢固树立以人民为中心的发展思想，建立完善正向激励机制和容错纠错机制，激发广大干部勇于担当、干事创业。增强营商环境就是生产力、竞争力的意识，坚持不懈地优化发展环境，带着责任和感情当好企业和群众的服务员，切实做到“有求必应、无事不扰”。建立健全工作督查机制，严格政府工作考核督查，按照目标明确、措施明确、责任明确的要求，推动各项工作具体到单位、实化到项目、量化到指标。突出问题导向，坚持深入一线、深入基层，做到及时主动发现问题、解决问题，确保重大政策、重大项目、重大工程落地见效。以政府机构改革为新起点，担当作为、奋力拼搏，开创各项工作新局面。

　　（五）强化廉政建设，树立新形象。严格落实全面从严治党主体责任和“一岗双责”，健全行政权力监督制约机制，以反腐败永远在路上的坚韧和执着，保持惩治腐败高压态势。严格贯彻落实中央八项规定精神及其实施细则，坚决纠正形式主义、官僚主义顽症，防止“四风”问题反弹。严格用制度管人、管权、管事，加强公共资金、国有资产、国有资源和领导干部经济责任审计监督，不断扎牢“不能腐”的笼子。坚持将纪律和规矩挺在前面，严格执行廉洁准则和纪律处分条例，加大群众身边腐败和作风突出问题专项治理，严肃查处侵害群众利益行为，做到无禁区、全覆盖、零容忍，营造干部清正、政府清廉、政治清明的良好氛围。

　　各位代表，新时代引领新发展，新目标扬帆新征程。让我们更加紧密地团结在以习近平同志为核心的党中央周围，在中共菏泽市委的坚强领导下，紧紧依靠全市人民，不忘初心、牢记使命，锐意进取、扎实苦干，加快突破菏泽、跨越赶超步伐，为“决胜全面小康、实现后来居上”宏伟目标奋力书写无愧于时代、无愧于人民的精彩华章！
